# Effectiveness of Evidence‐Based Practice‐Based Mentor Nurse Training Program: A Quasi‐Experimental Controlled Study Design

**DOI:** 10.1111/wvn.70069

**Published:** 2025-08-21

**Authors:** Vesile Koçak, Selda Arslan, Muradiye Aldem Budak

**Affiliations:** ^1^ Department of Nursing Necmettin Erbakan University Konya Turkey

**Keywords:** evidence‐based practice, mentor, nursing, nursing education, quasi‐experiment

## Abstract

**Background:**

The translation of evidence‐based practice (EBP) into routine nursing practice remains a persistent challenge.

**Aim:**

To evaluate the impact of a structured EBP Mentor Nurse Training Program, developed using the Johns Hopkins EBP model as a process guide and conceptually grounded in the ARCC (Advancing Research and Clinical Practice through Close Collaboration) model, which emphasizes the development of EBP mentors to enhance implementation and competency.

**Method:**

This quasi‐experiment used a pretest‐posttest design with equivalent control and intervention groups (*n* = 52; intervention group = 26, control group = 26). The intervention consisted of a blended training program (16 h face‐to‐face, 3 h online) covering EBP, mentoring, and communication skills. The control group received no intervention. Data were collected using the Nurse Information Form, the Evidence‐Based Practice Evaluation Competency Scale, and the Mentoring Scale. The TREND statement guided reporting.

**Results:**

Post‐test results indicated significant improvements in the intervention group's EBP knowledge sub‐dimension and total competency scores. Statistically significant gains were also observed in the coaching, role modeling, counseling, acceptance and approval, and friendship sub‐dimensions of the Mentoring Scale. Effect sizes ranged from *d* = 0.5 (coaching) to *d* = 0.8 (EBP knowledge), indicating moderate to large practical significance.

**Linking Evidence to Action:**

Structured EBP mentorship programs effectively enhance nurses' knowledge, EBP competencies, and mentoring abilities. These outcomes align with the ARCC model, supporting the cultivation of EBP mentors as a sustainable strategy for EBP integration. Incorporating blended learning and active mentorship in nursing education can foster a culture of collaboration, improve clinical decision‐making, and promote better patient outcomes.

## 1 Introduction

Evidence‐Based Practice (EBP) is an essential component of health care, integrating the best available scientific evidence, clinical expertise, patient preferences, and contextual factors to inform clinical decision‐making and enhance patient outcomes (Melnyk and Fineout‐Overholt 2019). Despite its well‐established efficacy in enhancing care quality and lowering costs (Connor et al. 2023), the translation of EBP into routine nursing practice remains a persistent challenge, particularly in environments where institutional infrastructure and staff capacity for EBP are limited (Leal‐Costa et al. 2024). To address these barriers, structured models, such as the Advancing Research and Clinical Practice through Close Collaboration (ARCC) model and the Johns Hopkins Evidence‐Based Practice (JHNEBP) model have been developed to guide both the cultural transformation of healthcare systems and the stepwise application of EBP in clinical decision making (Melnyk et al. 2004; Speroni et al. 2020).

Mentorship, a central strategy of the ARCC model, supports the application of EBP by strengthening nurses' confidence and competence in evidence‐based care. Its role in professional development and clinical integration is well recognized in nursing education and practice (Kallerhult Hermansson et al. 2024; Keinänen et al. 2023; Kowalski 2019). Building on these frameworks, this study aimed to develop and evaluate the effectiveness of a structured EBP Mentor Nurse Training Program designed to enhance nurses' competencies in EBP and mentoring. Guided conceptually by the ARCC model and procedurally by the JHNEBP model, the program was evaluated using a quasi‐experiment to assess its impact on EBP‐related beliefs, skills, and mentoring capacity.

## 2 Background

The ARCC Model is an evidence‐based system‐wide model for advancing and sustaining EBP in hospitals and healthcare systems (Saunders and Vehviläinen‐Julkunen 2017; Melnyk and Fineout‐Overholt 2023). The ARCC model offers a comprehensive, system‐level framework for implementing and sustaining EBP within healthcare organizations, serving as a strategic roadmap for assessing organizational readiness and developing EBP mentors to work with point‐of‐care nurses and clinicians in implementing EBP (Melnyk and Fineout‐Overholt 2023; Melnyk et al. 2011, 2018). It begins by evaluating the organizational culture for EBP, as well as clinicians' beliefs and current implementation levels, to identify both facilitators and barriers. A central component of the model is the intentional development of a critical mass of EBP mentors (Chays‐Amania et al. 2025; Melnyk and Fineout‐Overholt 2023). These mentors are clinicians with advanced EBP competencies who support point‐of‐care staff in translating evidence into practice and facilitating change at both individual and organizational levels (Melnyk et al. 2021). Research has demonstrated that the presence of EBP mentors and a strong EBP culture are associated with enhanced clinician competency, greater implementation of evidence‐based interventions, improved patient outcomes, and increased staff retention (Melnyk et al. 2010; Stokke et al. 2014; Wallen et al. 2010). Thus, the ARCC Model supports both professional development and institutional quality and sustainability in EBP delivery (Melnyk et al. 2017, 2021).

While the ARCC model provides the conceptual and system‐level foundation for this study, it is complemented by the JHNEBP model, which offers a stepwise framework for the practical implementation of EBP at the individual level. The JHNEBP model consists of three phases that guide clinicians in identifying clinical problems, evaluating evidence, and applying findings in practice, with a focus on stakeholder engagement, sustainability, and outcome evaluation (Bissett et al. 2025; Speroni et al. 2020). In this study, the ARCC model informed the conceptual framework and mentoring strategy, while the JHNEBP model guided the instructional content of the training intervention.

Mentorship has long been recognized as a foundational element of nursing education and professional development. In clinical settings, it plays a critical role in supporting the transition from theoretical knowledge to applied practice, while fostering confidence, communication, and leadership skills (Keinänen et al. 2023; Kowalski 2019). Mentoring is a dynamic and positive relationship that provides emotional support, socialization, and supports professional development (Kallerhult Hermansson et al. 2024). Furthermore, mentoring education facilitates learner‐centered assessment, individualized goal‐setting, and professional growth within a constructive pedagogical environment (Keinänen et al. 2023; Mikkonen et al. 2022). The need for mentoring‐specific training for healthcare professionals has been well documented, particularly in the context of enhancing mentoring capacity, improving educational outcomes, and supporting novice practitioners (Keinänen et al. 2023; Koole et al. 2016). Given this critical need, the present study was designed to evaluate the effectiveness of a structured Evidence‐Based Practice Mentor Nurse Training Program, developed in collaboration with academic experts and informed by the ARCC Model as the conceptual framework and the JHNEBP model as the process guide.

### 2.1 Study Aims

This study aimed to evaluate the effectiveness of an EBP‐Based Mentor Nurse Training Program, developed conceptually based on the ARCC model and procedurally guided by the Johns Hopkins EBP model, in improving nurses' evidence‐based practice competencies and mentoring skills.

## 3 Methods

### 3.1 Study Design

The study employed a quasi‐experimental pretest‐posttest design with a control group comprising similar participants. It adhered to the TREND (i.e., Transparent Reporting of Evaluations with Nonrandomized Designs) reporting guidelines for nonrandomized or quasi‐experimental study designs (Supporting Information).

### 3.2 Sample Size and Participants

**FIGURE 1:**
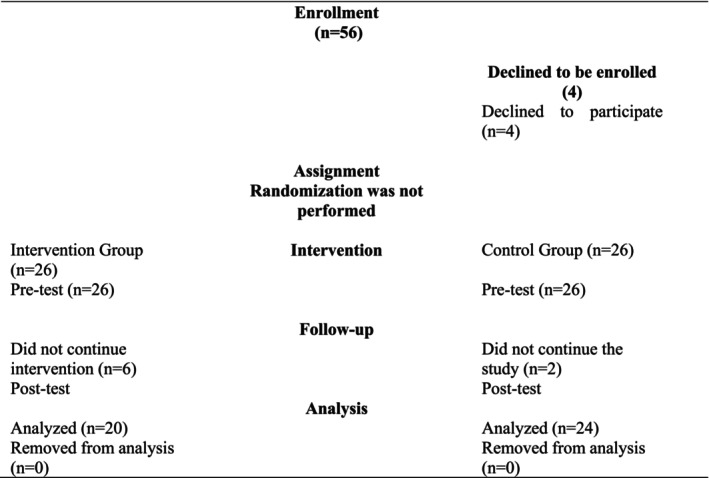
TREND flow diagram.

The training, limited to 30 participants within the scope of a project, was announced via the institution's website. Volunteer nurses who expressed interest in participating in the “Mentor Nurse” training constituted the intervention group (*n* = 26) (Figure 1). Due to the nature of this educational intervention study, randomization was not feasible for group allocation. Following the completion of registrations for the intervention group, an equal number of nurses were assigned to the control group. For the control group, nurses were randomly selected from the same institutions as those of the intervention group, ensuring comparability in terms of gender and educational level (*n* = 26). A total of 52 nurses participated in the study. Inclusion criteria encompassed holding at least a bachelor's degree in nursing, preferably a master's degree, and possessing a minimum of one year of professional experience. Exclusion criteria included non‐attendance at the training sessions and the presence of communication barriers, such as inability to speak Turkish, visual or auditory impairments, or diagnosed mental health or psychological conditions.

### 3.3 Data Collection

Data were collected online by the researchers from nurses who consented to participate in the study. Pre‐tests were administered to both the intervention and control groups prior to the commencement of the training program, and post‐tests were conducted following the completion of the training.

### 3.4 Intervention Group

**FIGURE 2:**
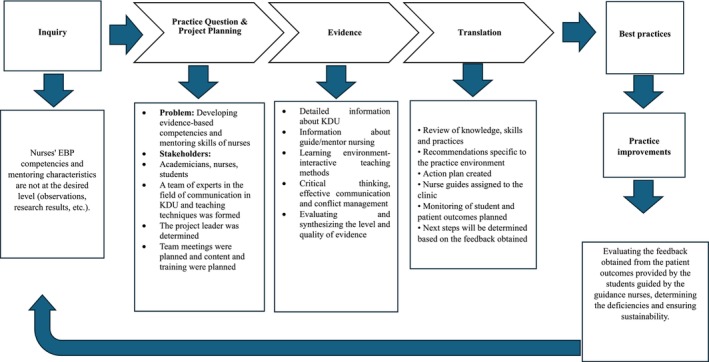
The Evidence‐Based Practice‐Based Mentor Nurse Training Program, developed according to the Johns Hopkins EBP model.

The study was conceptually grounded in the ARCC Model, which emphasizes building a critical mass of EBP mentors to improve EBP adoption across healthcare systems. In parallel, the JHNEBP model served as the process model guiding the practical implementation of EBP within the training curriculum (Figure 2). The JHNEBP model follows a structured three‐step approach: practice question, evidence, and translation to assist clinicians in identifying clinical issues, appraising evidence, and integrating findings into practice through feasible action plans and outcome evaluation.

Building on these models, the Evidence‐Based Practice‐Based Mentor Nurse Training Program was designed to enhance nurses' competencies in both EBP and mentoring. The program consisted of three sessions. The first session was delivered in‐person over two consecutive days, totaling 16 h, and included both theoretical instruction and practical activities focused on mentoring skills. A team of 12 academic experts in fields such as evidence‐based care, leadership, education, communication, and conflict resolution facilitated the training using diverse teaching methods such as lectures, group work, demonstrations, and interactive discussions. A blended learning format was adopted, integrating face‐to‐face and online sessions to enhance accessibility and engagement. Two months after the initial training, two follow‐up sessions were conducted online, each lasting approximately 90 min, to reinforce learning and maintain participant engagement.

### 3.5 Training Content of the Evidence‐Based Practice‐Based Mentor Nurse Training Program

The training program, developed in alignment with the JHNEBP model, was delivered in three sessions; combining both face‐to‐face and online formats.

#### 3.5.1 Session 1

This session focused on foundational mentoring and professional competencies. Topics included:

- Introduction to mentor nursing: roles, responsibilities, and essential characteristics of mentors.
- Creating a supportive and healing learning environment.
- Active learning strategies applicable in clinical settings.
- Clinical assessment and evaluation methods.
- Principles of measurement and evaluation.
- Effective communication skills and conflict management.
- Teamwork and collaboration.
- Legal and ethical issues in clinical nursing practice.
- Research literacy and evidence‐based practice in nursing.
- Application of the nursing process.
- Contemporary approaches and innovations in nursing care.

#### 3.5.2 Session 2

This session aimed to enhance nurses' cognitive and clinical decision‐making abilities, focusing on:

- Critical thinking and critical decision‐making in clinical settings.

#### 3.5.3 Session 3

The final session concentrated on strengthening evidence‐based practice skills:

- Evidence‐based practice definition and process.
- Selecting and using databases to access evidence‐based nursing information.
- Examples of evidence‐based nursing practice.

### 3.6 Control Group

No intervention was implemented for the nurses in the control group. As the training is scheduled to recur annually, nurses in the control group will have the opportunity to participate in future sessions. An announcement will be made to inform them once preparations for the upcoming training are finalized.

### 3.7 Hypotheses

*There will be a statistically significant difference between the intervention and control groups in the post‐intervention mean scores on the Evidence‐Based Practice Evaluation Competency*.

*There will be a statistically significant difference between the intervention and control groups in the post‐intervention mean scores on the Mentoring Scale*.

### 3.8 Data Collection Tools

Data collection tools used were the Nurse Information Form, Evidence‐Based Practice Evaluation Competence Questionnaire, and Mentoring Scale.

#### 3.8.1 Nurse Information Form

This form consists of a total of 5 questions regarding the age, gender, working years, educational status, and marital status of nurses.

#### 3.8.2 Evidence‐Based Practice Evaluation Competence Questionnaire (EBP‐COQ‐T)

The scale was developed by Ruzafa‐Martinez et al. (2013). The Turkish validity and reliability of the scale were conducted by Yildiz and Güngörmüş (2016). The scale consists of 3 sub‐dimensions (Factor 1: Knowledge; Factor 2: Skill; Factor 3: Attitude) and 25 items. It is a 5‐point Likert‐type scale. The lowest score that can be obtained from the scale is 25, and the highest score is 125. Negative items are reverse scored. The Cronbach's alpha coefficient of the scale was found to be 0.826.

#### 3.8.3 Mentoring Scale

The Turkish validity‐reliability study of the scale developed by Noe (1988) was conducted by Özkalp et al. (2006). The scale consists of 6 sub‐dimensions and 30 items. It is a 5‐point Likert‐type scale. The scale includes the sub‐dimensions of Coaching, Role Modeling, Self‐Expression and Visibility, Consultancy, Acceptance and Approval, and Friendship. The lowest score is 30, and the highest is 150. The Cronbach's alpha value of the scale is 0.96.

### 3.9 Statistical Analysis

The data were analyzed using IBM SPSS Statistics Standard Concurrent User V26 (IBM Corp., Armonk, NY, USA). Descriptive statistics (frequency, percentage, mean, standard deviation, median, interquartile range) were computed for numerical variables. The normality of the data was assessed using the Shapiro–Wilk test. For intergroup comparisons, the Mann–Whitney *U* test, independent *t*‐test, Pearson chi‐square test, and Yates' corrected chi‐square test were employed. A *p*‐value of < 0.05 was considered statistically significant. Additionally, effect sizes were calculated using Cohen's *d* to assess the magnitude of differences between groups.

### 3.10 Ethical Consideration

Ethics committee approval was obtained from the Ethics Committee of Necmettin Erbakan University, Faculty of Health Sciences Scientific Research Ethics Committee with approval number: 2023/586 to conduct the study. Informed consent was obtained from nurses who volunteered to participate in the study. The study was conducted in accordance with the Declaration of Helsinki. Participants were verbally informed about the study, and their informed consent was obtained. They were made aware that they could withdraw from the study at any time without providing a reason and that their participation would incur no costs. The study adhered to the principles of the Declaration of Helsinki.

## 4 Results

A comparison of the descriptive characteristics of participants in the intervention and control groups is presented in Table 1. Statistical analysis revealed no significant differences between the groups concerning these characteristics (*p* > 0.05) (Table 1). In the pre‐test measurements, a significant difference was observed between the intervention and control groups in the attitude sub‐dimension of the Evidence‐based Practice Evaluation Competence Questionnaire (*p* < 0.05). However, no significant differences were found in the other sub‐dimensions or the total score (*p* > 0.05). Post‐test measurements indicated a significant increase in the knowledge sub‐dimension and the total score of the Evidence‐based Practice Evaluation Competence Questionnaire in the intervention group (*p* < 0.05), as shown in Table 2. Furthermore, effect size analyses revealed that the intervention had a very large impact on the knowledge sub‐dimension (Cohen's *d* = 1.211) and a large effect on the total score (Cohen's *d* = −0.884) in the post‐test. These findings suggest that the intervention was particularly effective in enhancing participants' knowledge and overall competence in evidence‐based practice. Consequently, the H_a_ hypothesis was accepted. Regarding the Mentoring Scale, pre‐test measurements revealed a significant difference between the groups in the self‐expression sub‐dimension (*p* < 0.05), with no significant differences in the other sub‐dimensions or the total score (*p* > 0.05) (Table 3). In the post‐test measurements, significant differences emerged between the groups in the coaching (*p* = 0.045, Cohen's *d* = 0.626), role modeling (*p* = 0.028, Cohen's *d* = 0.815), consultancy (*p* = 0.025, Cohen's *d* = 0.686), acceptance and approval (*p* = 0.015, Cohen's *d* = 0.901), and friendship (*p* = 0.038, Cohen's *d* = 0.689) sub‐dimensions, as well as in the total score (*p* = 0.007, Cohen's *d* = 0.818). These effect sizes range from medium to large, indicating substantial improvements in the intervention group across multiple mentoring functions. Therefore, the H_b_ hypothesis was accepted.

**TABLE 1:** Demographic and baseline characteristics of the study participants (*n* = 52).

	Intervention group (*n* = 26)	Control group (*n* = 26)	Statistics	*p*
Characters of participants				
Participants age, mean (SD)	31.08 (5.24)	32.96 (8.67)	0.949^a^	0.347
Participants gender, *n* (%)				
Male	9 (34.6)	3 (11.5)	2.708^b^	0,100
Female	17 (65.4)	23 (88.5)		
Education status, *n* (%)				
University	8 (30.8)	8 (30.8)	0.000^c^	1.000
Master	15 (57.7)	15 (57.7)		
PhD	3 (11.5)	3 (11.5)		
Marital status, *n* (%)				
Married	14 (53.8)	15 (57.7)	0.000^b^	1.000
Single	12 (46.2)	11 (42.3)		
Working age, mean (SD)	6.96 (4.75)	9.98 (8.34)	1.604^a^	0.117

Abbreviation: SD, standard deviation.

^a^ Student's *t*‐test.

^b^ Chi‐square test with Yates correction.

^c^ *X* ^2^ test.

**TABLE 2:** Comparison of evidence‐based practice evaluation competence questionnaire scores between the intervention and control groups (*n* = 44).

	Intervention group (*n* = 20)	Control group (*n* = 24)	Statistics	*p*	Cohen *d*
Attitude, median (IQR)					
Pre‐test	47.50 (26.25)	58.50 (10.75)	‐2.113^a^	0.035	−0.888
Post‐test	60.50 (8.25)	58 (11.75)	1.586^a^	0.113	0.623
Skill, mean (SD)					
Pre‐test	21.55 (5.31)	23,04 (3.97)	1.066^b^	0.293	−0.322
Post‐test	24.45 (3.80)	22.58 (3.60)	−1.670^b^	0.102	−0.507
Knowledge, mean (SD)					
Pre‐test	19.40 (5.34)	19.50 (4.83)	0.065^b^	0.948	−0.020
Post‐test	24.50 (4.39)	19.63 (3.69)	−4.000^b^	0.000	1.211
Total, mean (SD)					
Pre‐test	89.10 (21.18)	99.21 (12.19)	1.890^b^	0.069	−0.590
Post‐test	108.75 (11.22)	97.46 (13.92)	−2.921^b^	0.006	−0.884

Abbreviations: Cohen *d* = effect size; IQR, interquartile range; SD, standard deviation.

^a^ Mann–Whitney *U* test.

^b^ Student's *t*‐test.

**TABLE 3:** Comparison of mentoring scale scores between the intervention and control groups (*n* = 44).

	Intervention group (*n* = 20)	Control group (*n* = 24)	Statistics	*p*	Cohen *d*
Coaching, median (IQR)					
Pre‐test	19.50 (9.50)	23 (5)	−1.750^a^	0.080	−0.642
Post‐test	24 (4)	20.50 (5)	2.000^a^	0.045	0.626
Role modeling, median (IQR)					
Pre‐test	20 (8.25)	20 (6.50)	−0.024^a^	0.981	−0.036
Post‐test	21 (5)	19.50 (3.75)	2.198^a^	0.028	0.815
Self‐expression and visibility, median (IQR)					
Pre‐test	18 (10.50)	21 (5)	−2.301^a^	0.021	−0.819
Post‐test	24.50 (5)	20 (6)	1.761^a^	0.078	0.610
Consultancy, median (IQR)					
Pre‐test	36 (14.50)	40 (9)	−1.908^a^	0.056	−0.652
Post‐test	42 (8.50)	36 (7)	2.244^a^	0.025	0.686
Acceptance and approval, median (IQR)					
Pre‐test	12.50 (4)	13.50 (3)	−1.205^a^	0.228	−0.495
Post‐test	15 (1)	12.50 (3)	2.443^a^	0.015	0.901
Friendship, median (IQR)					
Pre‐test	11 (7.75)	12 (5)	−1.234^a^	0.217	−0.385
Post‐test	12.50 (4.75)	11 (3)	2.072^a^	0.038	0.689
Total, median (IQR)					
Pre‐test	114 (50.50)	127.50 (31.25)	−1.604^a^	0.109	−0.594
Post‐test	133 (25.50)	120 (26.50)	2.701^a^	0.007	0.818

Abbreviations: Cohen *d*, effect size; IQR, interquartile range.

^a^ Mann–Whitney *U* test.

## 5 Discussion

This study is the first in Turkey to evaluate the impact of a structured EBP Mentor Nurse Training Program, developed using the ARCC and JHNEBP models, on the EBP competency and mentoring skills of clinical nurses. The ARCC Model served as the conceptual framework, emphasizing the development of a critical mass of EBP mentors to improve institutional adoption of EBP. The JHNEBP model was used as the process model, providing a structured framework to guide EBP training content.

Following the intervention, a significant increase was observed in the total score of EBP competency (effect size = 0.884) and the EBP knowledge sub‐dimension (effect size = 1.211) in the intervention group. These findings are consistent with previous research demonstrating that targeted EBP training can improve nurses' knowledge and capacity to apply evidence in clinical decision‐making (Elhabashy et al. 2024; Kim and Jeong 2024; Koota et al. 2021; Sapri et al. 2022). The effectiveness of this intervention may be attributed to the structured content, the inclusion of expert academic trainers, and the blended learning format. As noted in the literature, blended educational strategies enhance knowledge transfer through active methods such as group discussions, case studies, and real‐time feedback. These strategies promote peer interaction and address individual learning needs without logistical barriers (Dos Portela Santos et al. 2022; Wu et al. 2020). The educational intervention in our study was delivered via a blended approach, combining face‐to‐face and online learning which promoted active engagement through case studies, group discussions, and real‐time feedback. This outcome aligns with previous findings indicating that multi‐dimensional education programs can effectively enhance clinical nurses' EBP beliefs, skills, and attitudes (Liu et al. 2021). Additionally, the instructors consisted of academics who are experts in their fields, well‐versed in teaching techniques, and who provide up‐to‐date information with active teaching methods throughout the training. In order to ensure EBP competence among nurses, it is important to provide continuous teaching support blended with effective educational strategies by expert trainers in the field.

However, no statistically significant improvement was found in the skills and attitudes sub‐dimensions. This finding is consistent with prior studies reporting that, although knowledge may increase following an intervention, changes in attitudes are often not observed in the short term. In many cases, attitude shifts require a longer duration and tend to emerge gradually over time (Koota et al. 2021; Leal‐Costa et al. 2024; Mudderman et al. 2020; Vaajoki et al. 2023). Attitude refers to an individual's way of thinking and feeling about something, which often manifests in behavior (Oxford dictionary). Attitude consists of three components: emotion, thought, and behavior (Rosenberg et al. 1960). Individuals' emotions and thoughts (cognitions) affect their behavior. Here too, there seems to be a mutual interaction. While a positive attitude supports the learning and skill development process, a negative attitude hinders this process (Bandura 1977). In this context, attitude change requires more time, repetition, and reinforcement. Previous experiences, institutional culture, and motivational factors also play a role (Tomotaki et al. 2020). Therefore, future EBP training programs should consider long‐term and longitudinal approaches to reinforce skills and foster meaningful attitude change.

Importantly, the study demonstrated that the training program significantly improved mentoring skills in the intervention group. Improvements were noted across multiple mentoring sub‐dimensions, including coaching, role modeling, counseling, acceptance and approval, and friendship. These findings are consistent with earlier research indicating that mentorship‐focused interventions contribute to nurses' professional growth, facilitate the dissemination of EBP, and promote cultural change within clinical settings (Chan et al. 2020; Mudderman et al. 2020; Zhang et al. 2016). These outcomes strongly support the ARCC Model's emphasis on building EBP mentorship capacity as a driver of implementation success and institutional transformation. Mentorship not only supports nurses in adopting EBP practices but also enables them to act as role models and trainers for others, thus creating a multiplier effect within the healthcare workforce.

Mentoring is increasingly recognized as a critical competency in both clinical and educational nursing domains. As highlighted in prior literature, mentoring enhances professional identity, commitment, and clinical reasoning (Mikkonen et al. 2020). In the study, it is seen that the educational intervention was effective in the mentoring characteristics of nurses. Mentoring training interventions are a crucial phenomenon in both theoretical and clinical aspects of developing nurses' mentoring skills (Keinänen et al. 2023; Wang et al. 2024). The educational intervention presented in the study was found to increase the mentoring skills of nurses; attention is drawn to the integration of educational topics that address and develop mentoring skills into EBP educational interventions.

### 5.1 Limitations

This study has several limitations. First, the intervention was implemented for the first time within a limited time frame and budget, which may have influenced the program's overall impact. Second, the training was delivered to a relatively small group of nurses (maximum 30 participants), as required by the funding constraints. Small sample sizes may lead to overestimated effect sizes and reduced statistical power, which can limit the generalizability of the findings. Third, although the blended learning approach was effective, the short follow‐up duration may not have been sufficient to capture meaningful changes in attitudes and skills. Future studies should consider larger sample sizes, longer‐term evaluations, and multicenter implementation to strengthen the evidence base for EBP mentorship programs.

### 5.2 Linking Evidence to Action

- Structured EBP mentorship programs effectively enhance nurses' knowledge, EBP competencies, and mentoring abilities.
- These outcomes align with the ARCC model, supporting the cultivation of EBP mentors as a sustainable strategy for EBP integration.
- Incorporating blended learning and active mentorship in nursing education can foster a culture of collaboration, improve clinical decision‐making, and promote better patient outcomes.

## 6 Conclusion

This study demonstrated that the EBP Mentor Nurse Training Program, conceptually grounded in the ARCC Model and guided procedurally by the JHNEBP model, significantly improved nurses' EBP knowledge and mentoring competencies. These findings support the ARCC Model's emphasis on building a critical mass of EBP mentors to promote evidence‐based care and foster cultural change within healthcare organizations. However, limited improvements were observed in EBP attitudes and skills, suggesting the need for long‐term strategies and reinforcement. Future EBP training programs should adopt engaging, sustainable, and learner‐centered approaches that cultivate both knowledge and attitudinal transformation to support the full integration of EBP in clinical settings.

## Ethics Statement

The name of the ethics committee: Ethics Committee of Necmettin Erbakan University, Faculty of Health Sciences Scientific Research Ethics Committee. The approval number: 2023/586. The date of approval: 01.11.2023.

## Conflicts of Interest

The authors declare no conflicts of interest.

## Supporting information


**Data S1:** wvn70069‐sup‐0001‐Supinfo.pdf.

## Data Availability

The data that support the findings of this study are available from the corresponding author upon reasonable request.
